# Conditional Knockout of the Menkes Disease Copper Transporter Demonstrates Its Critical Role in Embryogenesis

**DOI:** 10.1371/journal.pone.0043039

**Published:** 2012-08-10

**Authors:** Yanfang Wang, Sha Zhu, Gary A. Weisman, Jonathan D. Gitlin, Michael J. Petris

**Affiliations:** 1 Department of Biochemistry, University of Missouri, Columbia, Missouri, United States of America; 2 Department of Nutrition and Exercise Physiology, University of Missouri, Columbia, Missouri, United States of America; 3 The Christopher S. Bond Life Science Center, University of Missouri, Columbia, Missouri, United States of America; 4 Marine Biological Laboratory, Woods Hole, Massachusetts, United States of America; University of Louisville, United States of America

## Abstract

The transition metal, copper (Cu), is an enzymatic cofactor required for a wide range of biochemical processes. Its essentiality is demonstrated by Menkes disease, an X-linked copper deficiency disorder characterized by defects in nervous-, cardiovascular- and skeletal systems, and is caused by mutations in the ATP7A copper transporter. Certain *ATP7A* mutations also cause X-linked Spinal Muscular Atrophy type 3 (SMAX3), which is characterized by neuromuscular defects absent an underlying systemic copper deficiency. While an understanding of these *ATP7A*-related disorders would clearly benefit from an animal model that permits tissue-specific deletion of the *ATP7A* gene, no such model currently exists. In this study, we generated a floxed mouse model allowing the conditional deletion of the *Atp7a* gene using Cre recombinase. Global deletion of *Atp7a* resulted in morphological and vascular defects in hemizygous male embryos and death *in utero*. Heterozygous deletion in females resulted in a 50% reduction in live births and a high postnatal lethality. These studies demonstrate the essential role of the *Atp7a* gene in mouse embryonic development and establish a powerful model for understanding the tissue-specific roles of *ATP7A* in copper metabolism and disease.

## Introduction

The ability of copper to cycle between Cu^1+^ and Cu^2+^ oxidation states enables it to function as a cofactor within a host of enzymes that catalyze a wide range of biochemical reactions. These include cellular respiration (cytochrome oxidase), connective tissue formation (lysyl oxidase), catecholamine synthesis (dopamine β hydroxylase), pigmentation (tyrosinase) and neuropeptide processing (peptidylglycine α-amidating monooxygenase) [1]. Not surprisingly, copper deficiency during gestation results in pleiotropic defects ranging from early embryonic lethality to long term postnatal defects in nervous-, circulatory- and skeletal systems [2]. The consequences of copper deficiency in humans are illustrated by Menkes disease (OMIM 390400), a pediatric disorder of copper metabolism caused by mutations in the *ATP7A* gene (NM_000052). The affected protein, ATP7A, is a copper transporting P-type ATPase that is predominantly located in the distal compartments of the Golgi complex where it loads copper into the secretory pathway for incorporation into nascent polypeptides [3], [4]. The ATP7A protein also functions in cellular copper export, which is coupled to copper-stimulated trafficking of the protein into post-Golgi vesicles that fuse with the plasma membrane [3].

Menkes disease patients exhibit a wide spectrum of defects that are attributable to copper deficiency, including connective tissue malformation giving rise to hemorrhage, neurological symptoms such as mental retardation and seizures, and hypopigmentation due to defects in melanin synthesis [1]. Occipital horn syndrome (OMIM 304150) is another variant of this disorder in which connective tissue defects predominate with little or no neurological abnormality [5]. As an X-linked recessive trait, Menkes patients are typically male, although heterozygous female carriers may exhibit subtle phenotypes such as mosaic hypopigmentation due to variation in X-chromosome inactivation [6]. Recently, two missense mutations in the *ATP7A* gene were shown to cause X-linked distal spinal muscular atrophy type 3 (SMAX3; OMIM 300489), a degenerative neuromuscular disorder that typically affects patients in the second or third decade of life. Although in vitro studies suggest that SMAX3-causing mutations diminish ATP7A copper transport activity and localization, a systemic copper deficiency is lacking in SMAX3 patients suggesting that the motor neurons may be hypersensitive to subtle perturbations in copper homeostasis [1], [7].

The striking differences between Menkes disease, OHS and SMAX3, particularly in the affected organs, highlights the need to understand the functions of ATP7A at a tissue-specific level. However, there is currently no animal model that allows *ATP7A* deletion to be restricted to a given tissue. Whereas spontaneous mutations in the *Atp7a* gene have been identified in the collection of Mottled mice, named for the variegated coat color of heterozygous females, these mutants are limited in addressing the tissue-specific functions of the Atp7a protein due in part to the fact that *Atp7a* null mutations are embryonic lethal, as seen in the Dappled and Tohoku mutants, [8]–[10]. Thus, the only available mutants for study are those in which mutations only partially disrupt Atp7a protein activity (e.g., Brindled or Blotchy mice) [11]. In addition, since the *Atp7a* gene is ubiquitously expressed, certain phenotypes in the Mottled mice may be secondary to defects in copper homeostasis in other tissues. In this study, we describe the generation of an *Atp7a* “floxed” mouse model by homologous integration of Lox P sites into the sequences surrounding exon 11. Global deletion of the *Atp7a* gene in male hemizygotes using Cre recombinase resulted in embryonic lethality and a loss of Atp7a protein expression. These findings provide unequivocal evidence that *Atp7a* is required for embryogenesis and provide a powerful template for elucidating the spatiotemporal requirements for *Atp7a in vivo*.

## Results

### Generation of a Floxed *Atp7a* Mouse Model

**Figure 1:**
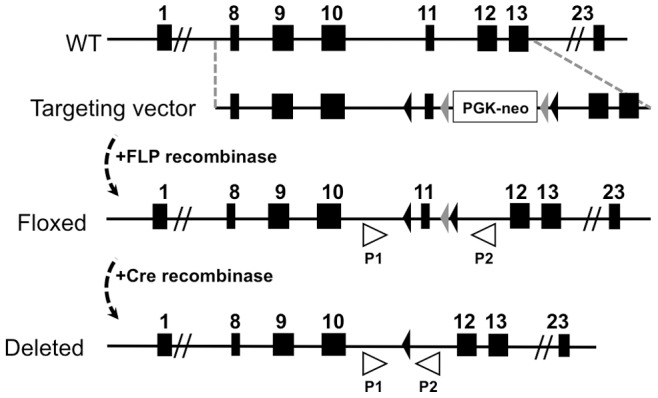
Schematic representation of the Atp7a gene-targeting strategy. Exons 8–13 of the wild type and recombinant *Atp7a* locus are shown. The targeting construct included LoxP sites (black triangles) that flanked exon 11 and a PGKneo gene cassette flanked by FRT sites (grey triangles). The PGKneo gene cassette was removed by crossbreeding with the ACT-FLPe deleter strain expressing FLP1 recombinase to generate mice with the desired floxed allele (*Atp7a^fl^*). The exon 11-deleted *Atp7a* allele (Deleted) is depicted following excision by Cre recombinase, which was detected using PCR primers P1 and P2.

A targeting construct encompassing exons 8 to 13 of the *Atp7a* gene was generated with Lox P sites flanking exon 11. Since this exon encodes a region of Atp7a protein that is highly conserved across orthologues of different phyla, its deletion by Cre recombinase was predicted to result in a nonfunctional protein (**Figure** **1**). The targeting construct also contained a neomycin resistance cassette that was driven by the phosphoglycerate kinase 1 promoter and flanked by FLP recombination sites (**Figure** **1**). Recombinant embryonic stem cells with homologous integration of the targeting vector were microinjected into Balb/c blastocysts. The resulting chimeras with a high percentage black coat color were mated to wild-type C57BL6 mice to generate F1 offspring carrying the recombinant *Atp7a* allele. The neomycin cassette was then removed by cross breeding the F1 mice with C57BL6 mice expressing FLP recombinase to generate males and females harboring the floxed *Atp7a* allele, hereafter designated *Atp7a^fl^* (**Figure** **1**).

### Global knockout of the Atp7a gene

**Figure 2:**
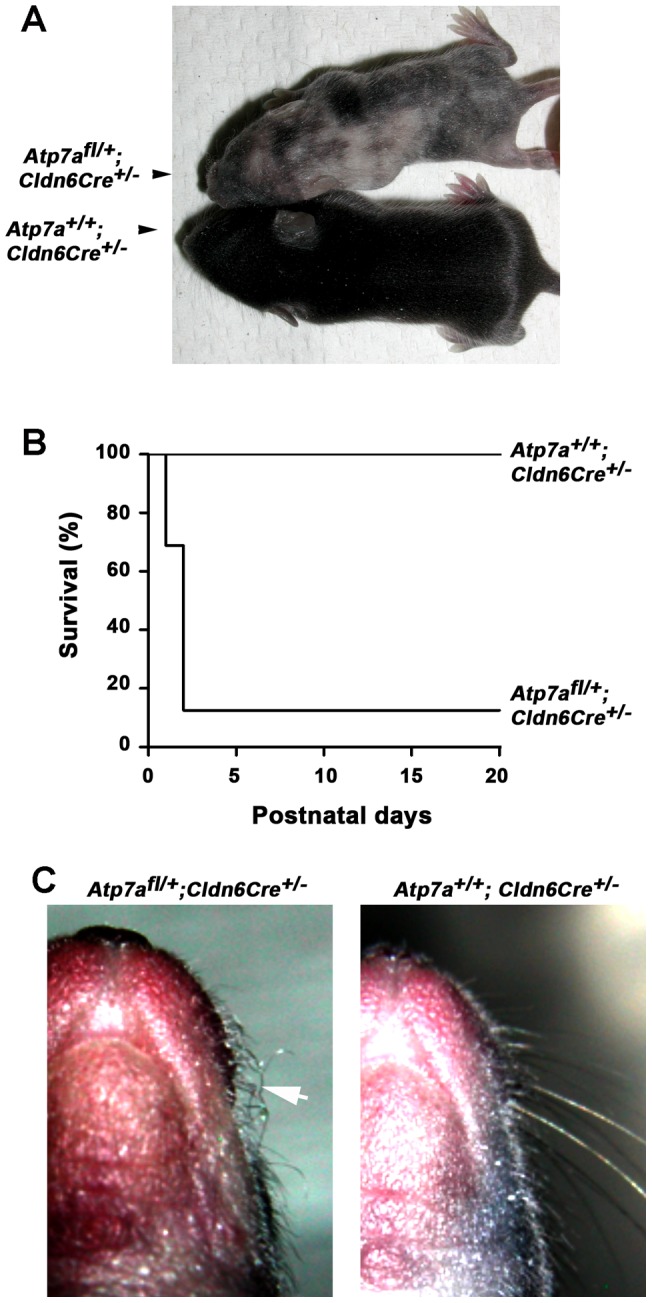
Characterization of *Atp7a^fl/+^*; *Clnd6Cre^+/−^* heterozygous female mice. A) Patches of hypopigmentation are observed in the coats of weanling heterozygous *Atp7a^fl/+^; Clnd6Cre^+/−^* females. B) Kaplan-Meier plot showing postnatal survival of heterozygous *Atp7a^fl/+^; Clnd6Cre^+/−^* female (n = 16) and littermate control *Atp7a^+/+^; Clnd6Cre^+/−^* (n = 34). C) Pili torti of the whiskers in surviving *Atp7a^fl/+^; Clnd6Cre^+/−^* females is shown (arrow).

To test whether Cre recombinase-mediated excision of exon 11 would generate a null allele of *Atp7a*, we crossed heterozygous floxed females (*Atp7a^fl/+^*) with mice that express Cre recombinase early in development throughout the epiblast by embryonic day 6.5 (E6.5) driven from the endogenous *Claudin 6* gene promoter (*Cldn6Cre^+/+^*). An absence of *Atp7a^fl/Y^; Cldn6Cre^+/−^* hemizygous male pups among a total of 80 live births suggested that this genotype was embryonic lethal (**Table** **1**), and was consistent with our finding of only half the expected ratio of *Atp7a^fl/+^; Cldn6Cre^+/−^* heterozygous females among these offspring. Of the 16 *Atp7a^fl/+^; Cldn6Cre^+/−^* female pups, only two lived beyond the second day of birth and these survivors exhibited a mosaic pattern of hypopigmented coat coloring (**Figure** **2A and 2B**), which was reminiscent of the variable pattern of X-chromosome inactivation that gives rise to the variegated coat color in heterozygous female carriers of Mottled mutations [12]. These females also exhibited curly whiskers (pili torti) (**Figure** **2C**), a well-documented feature of copper deficiency in the male hemizygous Mottled mutants [13]. Taken together, these results suggest that global excision of exon 11 of the *Atp7a* gene results in embryonic lethality in male hemizygotes, and a partially dominant haploinsufficiency in heterozygous females.

**Table 1:** Frequency of offspring with global deletion of the *Atp7a* gene.

Genotype	Live births	E10.5	Expected
***Atp7a^+/Y^; Cldn6Cre^+/−^***	30	17	25%
***Atp7a^fl/Y^; Cldn6Cre^+/−^***	0	13	25%
***Atp7a^fl/+^; Cldn6Cre^+/−^***	16	14	25%
***Atp7a^+/+^; Cldn6Cre^+/−^***	34	20	25%
**Total**	80	64	100%

The numbers of live-born offspring or embryos at day 10.5 post coitus (E10.5) are shown for each genotype resulting from a cross between *Atp7a^fl/+^* females and *Atp7a^+/Y^; Cldn6Cre^+/+^* males.

**Figure 3:**
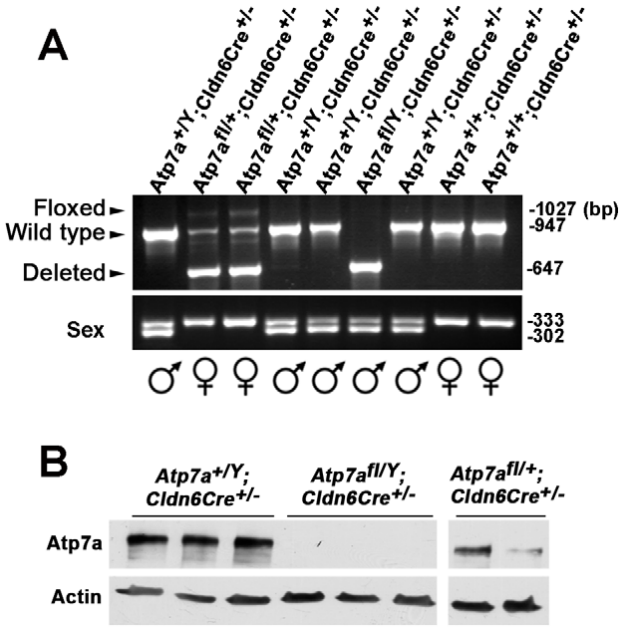
Exon 11 deletion in male hemizygous *Atp7a^fl/Y^*; *Clnd6Cre^+/−^* embryos. A) PCR analysis with P1 and P2 primers of yolk sac DNA of E10.5 embryos generated by crossing heterozygous floxed female mice (*Atp7a^fl/+^*) with *Cldn6Cre* mice (*Atp7a^+/Y^; Cldn6Cre^+/+^*). Cre-mediated deletion of the floxed allele (floxed) was detected by the presence of a 647 bp PCR product (Deleted). Sex was determined by PCR amplification of yolk sac DNA to simultaneously detect the X-chromosome-specific *Jarid1c* gene (331 bp) and the Y-chromosome-specific *Jarid1d* gene (302 bp). B) Immunoblot detection of Atp7a protein in E10.5 embryos. Blots were probed with anti-Atp7a antibody (1∶5000) followed by HRP-conjugated anti-rabbit antibody (1∶5000). Actin protein was detected on the same blot as a loading control.

**Figure 4:**
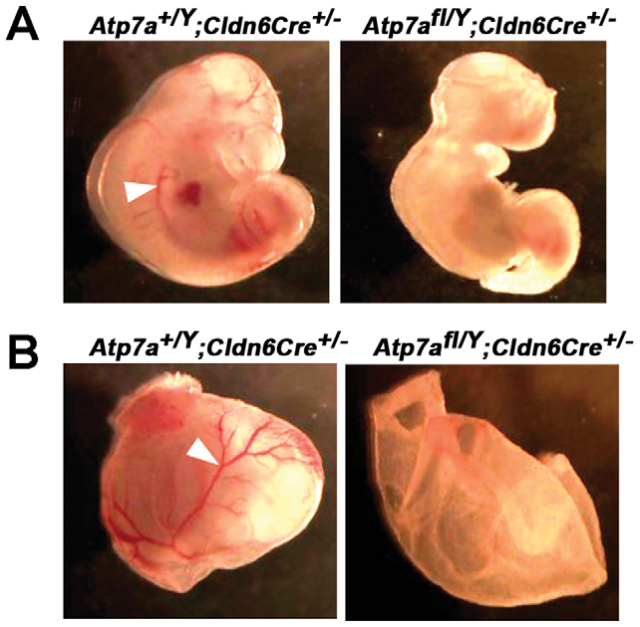
Morphological and vascular abnormalities in male hemizygous *Atp7a^fl/Y^*; *Clnd6Cre^+/−^* embryos. A) Developmental anomalies particularly in the frontonasal region and tail bud orientation were apparent in the *Atp7a^fl/Y^; Cldn6Cre^+/−^* embryos at E10.5. B) The blood-filled vascular network observed in wild type embryos and yolk sacs (arrowheads) was lacking for the *Atp7a^fl/Y^; Cldn6Cre^+/−^*.

To verify that the absence of Cre-positive floxed males among live births was not due to a failure of conception, embryos were collected and genotyped at E10.5. Of a total of 64 embryos analyzed, 13 were identified as Cre-positive floxed males (*Atp7a^fl/Y^; Cldn6Cre^+/−^*), which was close to the expected Mendelian ratio of 25% (**Table** **1**). PCR analysis of the yolk sac DNA confirmed highly efficient cleavage of exon 11 in the male hemizygous *Atp7a^fl/Y^; Cldn6Cre^+/−^* embryos, and the presence of both wild type and cleaved alleles in heterozygous *Atp7a^fl/+^; Cldn6Cre^+/−^* embryos (**Figure** **3A**). Immunoblot analysis demonstrated an absence of Atp7a protein in the male hemizygous *Atp7a^fl/Y^; Cldn6Cre^+/−^* embryos (**Figure** **3B**), and, as expected, the extent of this reduction was variable in *Atp7a^fl/+^; Cldn6Cre^+/−^* heterozygous female embryos (**Figure** **3B**). The E10.5 male knockout embryos were smaller than wild type sibling embryos, and exhibited striking morphological defects in the frontonasal region and the orientation of the tail bud (**Figure** **4A**). There was also a notable absence of a blood-filled vascular network in the knockout embryo and the surrounding yolk sac (**Figure** **4A and 4B**). These findings confirmed the essentiality of *Atp7a* in embryonic development and establish a conditional knockout mouse model of this gene.

### Characterization of wild type and knockout embryonic fibroblasts

**Figure 5:**
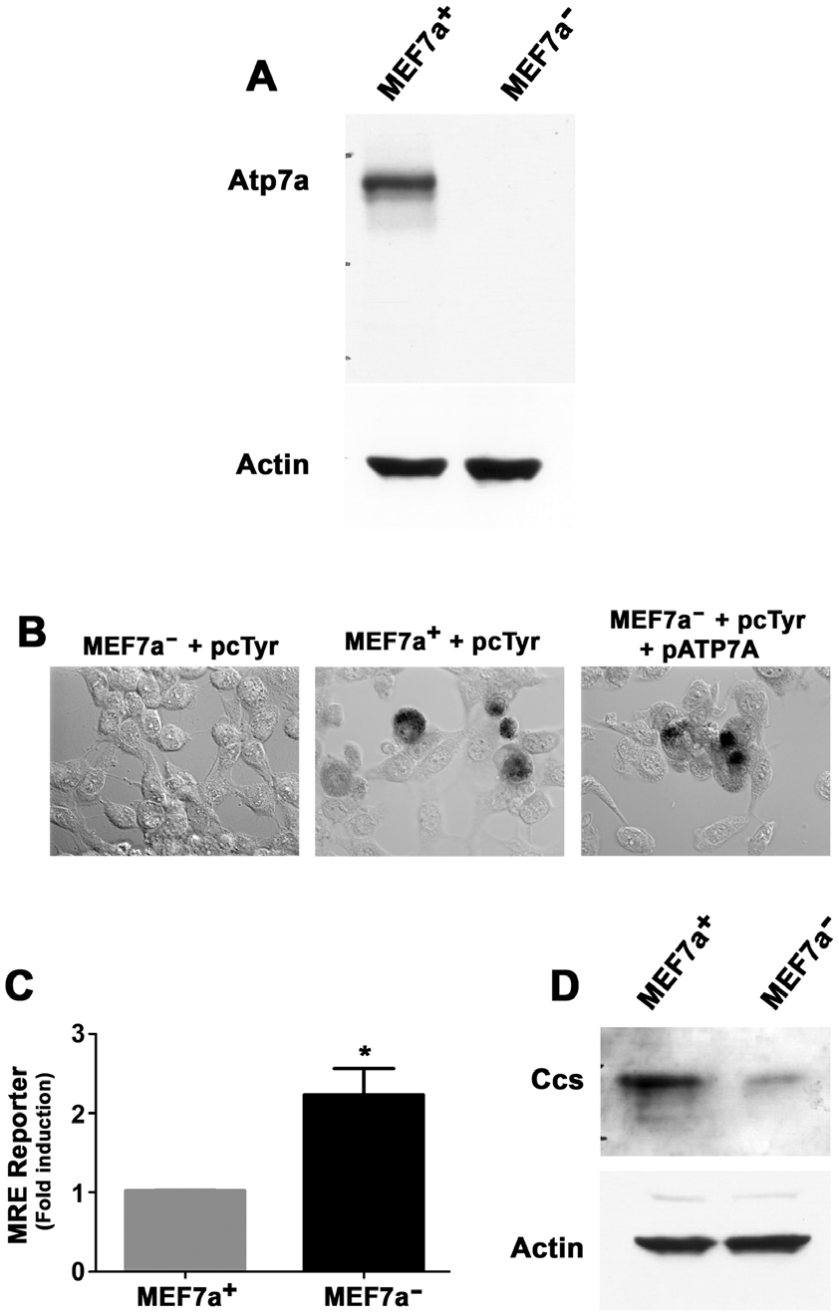
Characterization of MEF7a^+^ and MEF7a^−^ cells. A) Immunoblot analysis of Atp7a protein in MEF7a^+^ and MEF7a^−^ cells. Blots were probed with anti-Atp7a antibody (1∶5000) followed by HRP-conjugated anti-rabbit antibody (1∶5000). B) Defective copper delivery to tyrosinase MEF7a^−^ cells. MEF7a^+^ and MEF7a^−^ cells were transiently transfected with a plasmid encoding tyrosinase (pcTYR) alone, or in combination with a plasmid encoding ATP7A (pATP7A). Cells were then fixed in methanol and incubated with L-DOPA to allow the formation of the brown pigment, DOPAchrome. Note the absence of tyrosinase activity in MEF7a^−^ cells transfected with pcTYR alone (left), and complementation of this defect by co-transfection of pcTYR and pATP7A (right). C) MEF7a^+^ and MEF7a^−^ cells were transiently transfected with a Metal Responsive Element (MRE) reporter plasmid and a Renilla expression plasmid (pRL) as an internal control. After 24 hours, MRE luciferase activities of cell lysates were measured and normalized for Renilla luciferase activities. Experiments were repeated at least three times and values are presented as fold induction ± SEM; *p<0.05. D). Immunoblot analysis of Ccs protein in MEF7a^+^ and MEF7a^−^ cells. Blots were probed with anti-CCs antibody (1∶500) followed by HRP-conjugated anti-rabbit antibody (1∶5000).

Cell clones in which the presence or absence of a given protein is the sole genetic variable can often provide a powerful *in vitro* model for understanding its cellular function. To develop such a model for ATP7A, wild type mouse embryonic fibroblasts were obtained from E13.5 *Atp7a^fl/Y^* floxed male embryos and then immortalized by transfection of a plasmid encoding the SV40 large T antigen. A single clone, MEF7a^+^, was obtained after neomycin selection in which the Atp7a protein was detected by immunoblotting (**Figure** **5A**). Stable MEF7a^−^ derivatives of this cell line lacking expression of the Atp7a protein were cloned by transient transfection of MEF7a^+^ cells with a plasmid encoding Cre recombinase to excise Exon 11 of the *Atp7a* gene (**Figure** **5A**). Since Atp7a mediates the transport of copper from the cytoplasm into the Golgi, we investigated whether this function was lacking in MEF7a^−^ cells. Transfection of a plasmid encoding tyrosinase, an enzyme that requires copper loading within the secretory pathway via the Atp7a protein [14], resulted in the production of the DOPAchrome pigment in MEF7a^+^ cells, but not in MEF7a^−^ cells (**Figure** **5B**). The failure to produce DOPAchrome in MEF7a^−^ cells was due to a lack of Atp7a-mediated copper delivery to tyrosinase because DOPAchrome was produced when MEF7a^−^ cells were co-transfected with both tyrosinase and ATP7A expression plasmids (**Figure** **5B**). Additional studies demonstrated that the activity of a copper-inducible luciferase reporter plasmid was higher following transfection of MEF7a^−^ cells relative to MEF7a^+^ cells, consistent with higher cytoplasmic copper levels resulting from the loss of Atp7a protein (**Figure** **5C**). This was further supported by the finding of lower levels of the Ccs protein in MEF7a^−^ cells relative to MEF7a^+^ cells **(** **Figure** **5D**), a protein whose abundance is inversely proportional to copper concentration [15], [16].

## Discussion

The striking morphological and vascular defects observed in *Atp7a^fl/Y^; Cldn6Cre^+/−^* embryos at E10.5 resembled the phenotype of the most severe Mottled mutant in which embryonic lethality occurs at a similar stage of gestation [10]. Thus, the results presented in this study provide conclusive evidence that global deletion of the *Atp7a* gene in mice results in embryonic lethality. Although the precise biochemical basis for embryonic lethality in mice lacking an intact *Atp7a* gene is unknown, the Atp7a protein is known to deliver copper to at least three copper-containing enzymes whose deletion has been shown to cause lethality during gestation or the perinatal period, including peptidyl-glycine alpha-amidating monooxygenase, dopamine beta hydroxylase and lysyl oxidase [17]–[19]. Thus, it is reasonable to conclude that the phenotype of *Atp7a^fl/Y^; Cldn6Cre^+/−^* embryos is likely to stem from hypoactivity of these enzymes, and possibly others that acquire copper via the secretory pathway. It was also interesting to note that while heterozygous deletion of *Atp7a* in our study generated a high rate of postnatal mortality, heterozygous Mottled females are viable and able to propagate even the embryonic lethal Mottled mutations [11]. We speculate that the more severe phenotype in our heterozygous females may be due to the C57BL6 mouse background on which *Atp7a* mutations are known to be more severe than on other inbred strains [10], [20].

The floxed mouse model and the approaches used in this study will provide a powerful tool for deciphering the physiological importance of Atp7a protein in a spatiotemporal context. As there are currently no effective treatments for Menkes disease, occipital horn syndrome or SMAX3, deciphering the phenotypic of consequences tissue-specific *Atp7a* deletion may lead to new therapies for these disorders. Questions that we hope to address with this new animal model include the extent to which the neurological abnormalities in Menkes disease arise from a lack of copper delivery from the blood into the brain, versus a lack of ATP7A within the brain itself. Understanding such distinctions may have important implications for the development of membrane-permeable copper complexes able to carry copper ions across the blood brain barrier [21], [22], or the design of viral vectors that direct the expression of an *ATP7A* transgene in specific cell types of the brain in which a deficiency of Atp7a protein generates for the most severe neurological defects [20]. Addressing the requirement for Atp7a in motor neurons may shed light on the neuromuscular abnormality in patients with SMAX3. Of particular interest is whether SMAX3-like motor defects arise from copper toxicity in the motor neuron or a reduced copper delivery to cuproenzymes of the secretory pathway.

Notwithstanding the importance of ATP7A in these genetic disorders, numerous studies have also implicated ATP7A in a wide range of highly specialized processes beyond cellular copper homeostasis including glutamateric signaling and synaptogenesis in neurons [23], [24], macrophage bactericidal activity [25], oxidation of low density lipoprotein particles [26], smooth muscle migration [27], Alzheimer's disease [28], and multidrug resistance in cancer cells [29], [30]. Thus, the ability to selectively delete *Atp7a* in a tissue-specific manner may provide new insights into the broader roles of copper in normal physiology and disease states.

## Materials and Methods

### Animals and ethics

All animal husbandry and euthanasia procedures were performed in accordance with and under the approval of the Animal Care and Use Committee of the University of Missouri. All mice were on the C57BL6 strain background. Mice were maintained on 12 hour light-dark cycle and food and water provided *ad libitum*. Picolab diet 5053 (13 ppm Cu) was provided to mice at weaning and Picolab diet 5058 (17 ppm Cu) was provided during pregnancy and lactation (PMI International, St. Louis, MO, USA).

### Generation of the *Atp7a* floxed mouse

Portions of the floxed mouse production were performed under contract by inGenious Targeting Laboratory, INC (Stonybrooke, NY). A 9.7 kb *Pvu*II restriction fragment from a bacterial artificial chromosome library of C57BL6 mouse genomic DNA (clone RPC1-23-186F4) encompassing exons 8 to 13 of the *Atp7a* gene was subcloned into the pSP72 vector (Promega). A 62 bp single loxP cassette was inserted at a unique KpnI site located 123 bp upstream of the 5′ splice boundary of exon 11. A neomycin cassette flanked by LoxP and FRT sites was excised from the plasmid pGK-gb2 loxP/FRT Neo (Gene Bridges GmbH, Germany) and inserted 313 bp downstream of the 3′ splice exon 11 at unique BamHI site. The targeting vector was confirmed by sequencing. Ten micrograms of the targeting vector was linearized by NotI digestion and electroporated into C57BL/6 embryonic stem cells (Taconic, Hudson, NY), and single clones were selected for resistance to G418. Cell clones containing homologous insertion of long and short arms of the targeting vector were confirmed by southern blot and PCR analysis, and sequencing across LoxP sites and exons was used to verify fidelity of integration. Five independent correctly targeted clones were microinjected into Balb/c blastocysts and resulting chimeras with a high percentage black coat color were mated to wild-type C57BL6 mice to generate F1 offspring. Removal of the neomycin cassette via excision of flanking FRT sites was achieved by cross-breeding to FLP recombinase-expressing mice (strain B6.Cg-Tg(ACTFLPe)9205 Dym/J; Jackson Laboratory, Bar Harbor, ME). Floxed mice lacking the Neo cassette were identified by PCR analysis of tail genomic DNA and maintained on the C57BL6J background.

### Conditional knockout of *Atp7a*

To generate the global knockout, *Atp7A^fl/+^* females were crossbred with *Cldn6^tm1(cre)Dkwu^*/J mice (Jackson Laboratory). Tail snips from offspring were genotyped using PCR primers P1 (5′- GACAATACTACACTGACCATATTCA -3′) and P2 (5′-GTTCCACAGAAACTATATGCCTGGG -3′). Detection of the Cre recombinase transgene was achieved using primers CreF (5′- GATCGCTGCCAGGATATACG -3′) and CreR (5′-AATCGC CATCTTCCAGCAG -3′). Timed pregnancy was established by confirmation of a copulation plug in female mice the morning after introduction to a breeding male, and this was designated as embryonic day E0.5 of pregnancy. At day E10.5, embryos were isolated from pregnant dams and the yolk sacs were separated for genotyping as described above. Sex determination was performed as described previously [31].

### Immunoblotting

Tissue samples and embryos were homogenized in ice-cold phosphate buffered saline (PBS) at pH 7.4, and protein lysates were prepared by sonicating cell pellets in lysis buffer containing 2.5 mM Tris–HCl (pH 7.4), 2% sodium dodecyl sulfate, 1%Triton X-100, 1 mM EDTA and Complete^TM^ protease inhibitor (Roche Molecular Biochemicals, Indianapolis, IN). Protein lysates were fractionated by 7.5% sodium dodecyl sulfate–polyacrylamide gel electrophoresis and transferred onto nitrocellulose membranes. The membranes were blocked with 1% casein solution and incubated in blocking buffer at 4°C overnight with a rabbit anti-Atp7a antibody, described previously [32], raised against the terminal 20 amino acids (1∶5000 dilution) or mouse β-actin antibody (1∶5000) (abcam, Cambridge, MA). Horseradish peroxidase conjugated goat anti-rabbit IgG or goat anti-mouse IgG (1∶5000) were used as secondary antibodies, and blots were developed using the SuperSignal West Pico Substrate according to the manufacturer's instructions (Pierce, Rockford, IL).

### Isolation and Culture of Mouse Embryonic Cells

Hemizygous male floxed embryos (*Atp7a^fl/Y^*) were dissected from the decidua at embryonic day 13.5 and all tissues remaining after removal of the head and internal organs were minced with scissors and then incubated in 0.25% trypsin at 37°C for 15 minutes to facilitate dispersal into a single cell suspension. The cells were then cultured in complete medium (Dulbecco's modified Eagle's medium supplemented with 10% fetal bovine serum, 2 mM glutamine and 100 units/ml penicillin and streptomycin). After 3 weeks of culture, the cells were passaged onto 100 mm dishes and transfected with the pSV3-neo plasmid carrying SV40 large T antigen gene using the LipoD293 transfection reagent (SignaGen, Rockville, MD), and selected for resistance to neomycin. Single colonies were then expanded to generate multiple immortalized MEF7a^+^ cell lines. To generate an isogenic cell line lacking Atp7a protein expression (MEF7a^−^ cells), the *Atp7a* gene was deleted by transient transfection of one MEF7a^+^ cell clone with a plasmid carrying the Cre recombinase gene fused to GFP (pCAG-Cre: GFP; Addgene, Cambridge, MA). Cells enriched for GFP expression were sorted into 96 well trays by fluorescence activated cell sorting, and single clones were expanded. Deletion of the *Atp7a* gene was confirmed by PCR genotyping.

### Luciferase Reporter Assay

MEF7a^+^ and MEF7a^−^ cells were seeded in 96-well plates and transiently transfected with 35 ng of the MRE-luciferase reporter [33], and 3.5 ng of Renilla luciferase plasmid (pRL) using LipoD293 transfection reagent (SignaGen Laboratories). After a 24-hour incubation in complete medium, the firefly luciferase activity and Renilla luciferase activity were assayed by luminometer (Veritas) using the Dual-Glo luciferase assay system (Promega, Madison, WI). The relative light units (RLU) were calculated by dividing firefly luciferase activity by Renilla luciferase activity. Fold induction was calculated as mean RLU produced by MEF7a^−^ cells divided by mean RLU produced by MEF7a^+^ cells.

### In Situ Tyrosinase Activity Assay

In situ tyrosinase activity was conducted in MEF cells as previously described by detecting the formation of brown DOPA-chrome from the oxidation of colorless L-DOPA [14].
